# News and Coming Events

**Published:** 1907-07-27

**Authors:** 


### NEWS AND COMING EVENTS.

The total amount received up to date on behalf of the Metropolitan Sunday Fund is about £43,000.

The annual Court of Governors of the North-West London Hospital, Kentish Town Road, N.W., will be held at the hospital on Wednesday, July 31, 1907, at five o'clock in the afternoon. The Right Hon. Lord Rathmore will preside. And at the same place on the same day, at 5.15 p.m., or so soon thereafter as the annual general court shall be concluded, a special Court of Governors will be held for the purpose of considering, and, if thought fit, approving a scheme of amalgamation of the North-West London Hospital with the Hampstead General Hospital.

Mr. Otto Beit has presented to the Royal National Orthopædic Hospital a quantity of orthopædic apparatus for the furnishing of a gymnasium for the hospital. The appliances are for the exercising of the various joints and muscles of the body, and are all of the well-known "Zander" make, and the fittings and materials are of the finest quality. The apparatus is now fixed and in use in the hospital's temporary premises in Bolsover Street, and it is likely to prove of great benefit to the patients of the institution. Mr. Beit has consented to become an honorary life governor of the hospital.

The growing popularity of caravaning as a pastime is so marked that a "Caravan Club of Great Britain and Ireland" has been formed, the objects of which are to bring together those interested in van-life as a pastime, to arrange camping grounds, as far as it is found practicable in various centres, and to further and protect the interest of amateur caravanists. The honorary secretaries of the Club are Mr. J. Harris Stone, 72 Stamford Brook Road, W., and Mr. J. C. B. Eeis, 23 Dartmouth Park Road, Highgate Road, N.W., from whom all particulars may be obtained on application.

At the bi-monthly meeting of the Board of Management of the Royal Victoria Hospital, Belfast, the Treasurer, Mr. Davidson, read a letter from the sub-committee of the Dufferin Memorial Fund, intimating that they had decided to use this money in naming a bed in the Royal Victoria Hospital. The Board accepted the gift with gratitude, and returned their most cordial thanks to the committee for the generous donation. The following is the inscription to be placed over the bed : "The Dufferin Bed. In memory of the Most Honourable the Marquess of Dufferin and Ava, K.P. 1826—1902. Dedicated by the many friends associated with the erection of his memorial statue in the city of Belfast."

On Saturday, July 13, the new wing at Bolingbroke Hospital, Wandsworth Common, was opened for inspection. The addition consists of one of the three blocks which, with an administrative block, will form the entire hospital. The building has accommodation for two house surgeons and five nurses, and contains an operating-room and wards for seventeen beds, besides offices and stores. In every detail the utmost care has been taken to render insanitation impossible. Natural ventilation is provided for throughout the building. The walls are tiled, all the woodwork is enamelled, and the flooring is polished. The roof is flat and has been asphalted; it will be used as a promenade for convalescents. The cost of the new building is about £25,000. A considerable sum is required to make up the total of £80,000 for the completion of the entire hospital.

A meeting was held on July 22 at Chelsea Infirmary to consider the advisability of forming an Association consisting of Poor-law infirmary matrons. It was agreed unanimously that an Association was urgently required. The constitution was drawn up, and it was decided to hold a quarterly meeting for the discussion of questions of interest.

The Lord Mayor and Corporation of Cardiff, in order to commemorate the recent visit of their Majesties and H.R.H. the Princess Victoria, intend to present 40,000 specially designed boxes containing milk chocolate to the school children of that city, and have entrusted the execution of this large order to Messrs. J. S. Fry and Sons, Ltd., of Bristol and London, makers to H.M. the King.

Arrangements for the amalgamation of the Royal National and City Orthopædic Hospitals have now been completed, and the patients of both hospitals are being seen in the temporary premises of the amalgamated institution, Nos. 45-47 Bolsover Street, immediately behind the old hospital in Great Portland Street. Dr. Hughlings Jackson has been elected honorary consulting physician to the hospital, and Dr. Sutherland, Mr. John Poland, and Mr. J Jackson Clarke have been elected honorary physician, honorary surgeon, and honorary surgeon respectively. The new building operations have been begun, and a hospital for 213 in-patients is being built on the site of the old hospital and the adjoining premises. The new out-patient department is to be separated from the hospital, and will be on the opposite side of the road, on the east side of Bolsover Street, the hospital and the out-patient department being connected by a subway across Bolsover Street. Temporary accommodation for in-patients is provided in three of the vacant wards at Charing Cross Hospital which have been rented by the Royal National.

The arrangements for the social side of the meeting of the British Medical Association at Exeter have not been neglected. On Tuesday afternoon the Mayor of Exeter will give a garden-party; on Wednesday afternoon garden-parties will be given by the President of the South-Western Branch, Dr. Deas, and by Dr. Samways; on Thursday afternoon Sir Thomas Dyke Acland, Bart., will give a garden-party, and Dr. Gidley, Cullompton, will entertain a picnic party at tea; on Friday afternoon the High Sheriff of Devon, who is also President of the Royal Devon and Exeter Hospital, Sir Dudley Duckworth King, Bart., will give a garden-party at Wear House; and there will be luncheon-parties at Teignmouth, Torquay, and Sidmouth. On Wednesday evening there will be a reception by the South-Western Branch on Northernhay, and on Friday evening the civic authorities will hold a reception in the Albert Memorial. The annual dinner of the Association will take place on Thursday evening, and on the same evening ladies accompanying members will be entertained at the theatre by the Ladies' Committee. On Saturday several long excursions have been arranged. One party will visit Plymouth, where the Mayor will entertain them at luncheon. In the afternoon Plymouth Sound and the River Tamar will be visited, and the party will be entertained at tea by the Earl of Mount Edgcumbe. Other parties will visit Falmouth and Ilfracombe; others Bideford and Clovelly, where they will be entertained by Mrs. Hamlyn, of Clovelly Court; and others Tavistock, driving thence to Endsleigh, where they will be entertained at luncheon by the Duke of Bedford.

